# Ultrasound-guided botulinum toxin type A for shoulder pain: a meta-analysis of randomized controlled trials

**DOI:** 10.1186/s12891-025-09347-8

**Published:** 2026-01-08

**Authors:** Shiying Zhuang, Xiaoli Zhang, Cheng Lin, Zhizhuo Wang

**Affiliations:** Department of Rehabilitation Medicine, School of Health, Fujian Medical University, No. 1 Xuefu North Road, University Town, Fuzhou, Fujian 350122 China

**Keywords:** Ultrasound-guided, BoNT-A, Injection, Shoulder pain, ROM, Upper limb function, Quality of life, Meta-analysis

## Abstract

**Background:**

Shoulder pain is a very common symptom. A number of studies have demonstrated that botulinum toxin type A is effective in relieving shoulder pain. Therefore, this systematic review and meta-analysis aimed to synthesize scientific evidence and quantify the combined effects of ultrasound-guided botulinum toxin type A on shoulder pain.

**Methods:**

A comprehensive literature search was conducted in databases such as PubMed, Embase, Scopus, Cochrane Central Register of Controlled Trials (CENTRAL), China National Knowledge Information Database (CNKI), Wanfang database, and VIP database (VIP) using the keywords "ultrasound", "Botulinum toxin type A", and "shoulder pain". Two reviewers independently reviewed the studies, extracted data from eligible studies, and assessed the risk of bias. A random-effects model was used to calculate the standardized mean difference (SMD) and 95% confidence interval (CI) for Visual Analog Scale (VAS), Upper Extremity Fugl-Meyer Assessment (UEFMA), Range of Motion (ROM), Modified Barthel Index (MBI). Funnel plots and sensitivity analyses were also employed to evaluate the four outcome indicators above.

**Results:**

Out of retrieved 854 records, ten studies (involving 533 patients) were finally included. Pooled analysis showed that ultrasound-guided botulinum toxin type A was associated with large improvements in shoulder pain (SMD = -1.1; 95% CI -1.47 to -0.73;* P* < 0.001), UEFMA score (SMD = 1.43; 95% CI 0.49 to 2.37; *P* = 0.003), ROM of shoulder flexion (SMD = 1.28; 95% CI 0.63 to 1.93; *P* < 0.001) and external rotation (SMD = 1.66; 95%CI, 0.83 to 2.48; *P* < 0.001). Mild improvements were observed in ROM of shoulder abduction (SMD = 0.8; 95%CI 0.18 to 1.43; *P* = 0.01) and MBI score (SMD = 1.33; 95% CI 0.22 to 2.43; *P* = 0.02).

**Conclusions:**

Our meta-analysis has shown ultrasound-guided BoNT-A injections have potential benefits for reducing shoulder pain and improving upper limb function, range of motion, and quality of life. However, these findings should be interpreted cautiously due to small sample size, measured differences, substantial heterogeneity and possible publication bias. More high-quality studies with large sample size are needed to assess long-term efficacy, strengthening the evidence that ultrasound-guided BoNT-A facilitates the reduction of shoulder pain.

**Supplementary Information:**

The online version contains supplementary material available at 10.1186/s12891-025-09347-8.

## Background

Shoulder pain demonstrates a substantial epidemiological burden, with point prevalence and over 12-month estimates as high as 26% and 52%, respectively [1]. In the United States, this condition imposes substantial socioeconomic costs, including an estimated $299–335 billion in productivity losses attributable to pain-related disabilities in 2010 [2]. Clinically, shoulder disease may lead to dysfunction of upper limb motor function and mobility, leading to depressive states and poor health-related quality of life [3]. Without proper intervention, negative functional performance further increases negative psychology and quality of survival, potentially progressing to severe disability and morbidity [4, 5]. Therefore, the treatment of shoulder pain is highly of importance.

Various therapeutic approaches for shoulder pain management involve physical therapy [6, 7], pharmacotherapy [8], intra-articular steroid injections [8], dexamethasone administration [9], and subscapular nerve block [10]. However, these treatments are not always effective, and some have even been associated with adverse effects [11, 12]. For instance, dexamethasone injections can induce transient hyperglycemia within 24 h [13], while steroid injections may impair tendon healing [14]. In addition, Lam et al. suspected that nerve block may be associated with a risk of nerve damage [15]. Overall, it is indispensable to find the safe and effective analgesic therapies. In recent years, botulinum toxin injection has been used for analgesia, with a systematic review demonstrating a mean reduction of 2.59 points on the visual analog scale (VAS) in patients with fascial pain [16]. Evidence from a meta-analysis on the effectiveness of botulinum toxin for shoulder pain treatment revealed that the experimental group experienced more effective pain relief and a greater increase in joint mobility [17]. A comparative randomized controlled trial in hemiplegic shoulder pain found that pectoralis major and teres major BoNT-A injections showed comparable short-term efficacy to suprascapular nerve block (SSNB), with superior medium-term outcomes [18]. Nevertheless, therapeutic responses to BoNT-A remain inconsistent across studies, as some randomized controlled trials have failed to demonstrate significant advantages over placebo for post-stroke shoulder pain [19–21]. The observed discrepancies in therapeutic outcomes may stem from three key considerations: (i) population differences (e.g., age range: 25–55 years [20] vs 18–75 years [18]), (ii) methodological variations (e.g., follow-up duration: 4 months [19] vs 6 weeks [15]), and (iii) technical factors (landmark-guided injections [21] vs ultrasound [18]). Specifically, age-related neuromuscular junction changes may affect toxin binding [22], and the shorter follow-up period failed to capture the complete treatment trajectory, including the waning phase [23]. Notably, the guidance technique represents a critical determinant, with ultrasound guidance offering superior precision through real-time visualization of target musculature and adjacent anatomical structures [24]. This imaging capability enhances needle placement accuracy, potentially optimizing therapeutic efficacy while minimizing adverse events associated with imprecise injections [25]. A trial comparing injection methods demonstrated that ultrasound-guided approaches provide superior precision for most shoulder areas compared to blind techniques, although both methods exhibited comparable efficacy for subacromial space injections [26]. Similarly, Bae et al. documented enhanced treatment outcomes (24 U BoNT-A in masseter muscles) with ultrasound guidance versus blind techniques [27]. In contrast, Rijs et al. [28] and Cole et al. [29] reported that there were no significant differences in pain between microinjections guided by anatomical landmarks and those performed under ultrasound guidance.

To date, several meta-analyses on the treatment of botulinum toxin have reached a consensus that botulinum toxin injections have beneficial effects on patients with shoulder pain. However, the clinical effectiveness of ultrasound-guided BoNT-A injections has not been fully proven. Based on a comprehensive search of PubMed, Cochrane Central Register of Controlled Trials (CENTRAL), Scopus, WanFang database, China National knowledge Information Database (CNKI) and VIP database through December 2023 without any language restrictions, no meta-analyses have summarized the efficacy of the BoNT-A on shoulder pain via ultrasound guidance so far. Thus, the current study aimed to synthesize the scientific evidence and quantify the pooled effect of the ultrasound-guided BoNT-A injection in individuals with shoulder pain.

## Method

A meta-analysis was conducted in accordance with the PRISMA (Preferred Reporting Items for Systematic Reviews and Meta-Analysis) declaration and the Cochrane Handbook [30]. Our protocol was registered in the Prospective Register of Systematic Reviews (Ref: CRD42023493074, available at: https://www.crd.york.ac.uk/PROSPERO/display_record.php?RecordID=493074).

### Inclusion and exclusion criteria

The inclusion criteria were defined according to PICOS guidelines [31]: (i) Participants (P): Patients with shoulder pain of diverse etiologies (post-stroke, myofascial, and mixed origins). This combined analysis is biologically plausible given the shared central sensitization biomarkers like interleukin-6 (IL-6) observed in both post-stroke and myofascial pain [32]. (ii) Intervention (I): Ultrasound-guided BoNT-A injections. The intervention standardization across etiologies is supported by ultrasound for precise targeting of anatomically consistent muscles regardless of pain origin [33]. (iii) Comparison (C): All relevant studies with placebo and other active control groups were included. (iv) Outcome (O): First, shoulder pain was measured by the VAS and/or simple McGill pain score. Second, shoulder function was assessed by the passive range of motion (PROM), upper extremity Fugl-Meyer assessment (UEFMA) and/or modified Ashworth scale (MAS). Third, quality of life was assessed by the quality of life (QoL) scale, and activities of daily living (ADL) ability was assessed by the modified Barthel index (MBI) score. Lastly, the therapeutic effect was evaluated for clinical efficacy evaluation. (v) Study design (S): Randomized controlled trials (RCTs).

We excluded the following studies: (i) non-RCT trials; (ii) studies without full text (such as posters and conference abstracts); (iii) unpublished or withdrawn studies; (iv) studies on animal experiments and (v) studies with unknown sample size.

### Search strategy

An extensive search strategy was used for the seven data sources, including PubMed, Cochrane Library, CENTRAL, Scopus, WanFang database, CNKI and VIP from database inception to December 2023 without any language restrictions. These databases were used to identify ultrasound-guided BoNT-A injections for shoulder pain of diverse etiologies. We used the following terms in various combinations: “ultrasound’’, “BoNT-A’’, “shoulder’’, “pain’’ (detailed in supplementary file 1).

### Study selection and data extraction

**Fig. 1:**
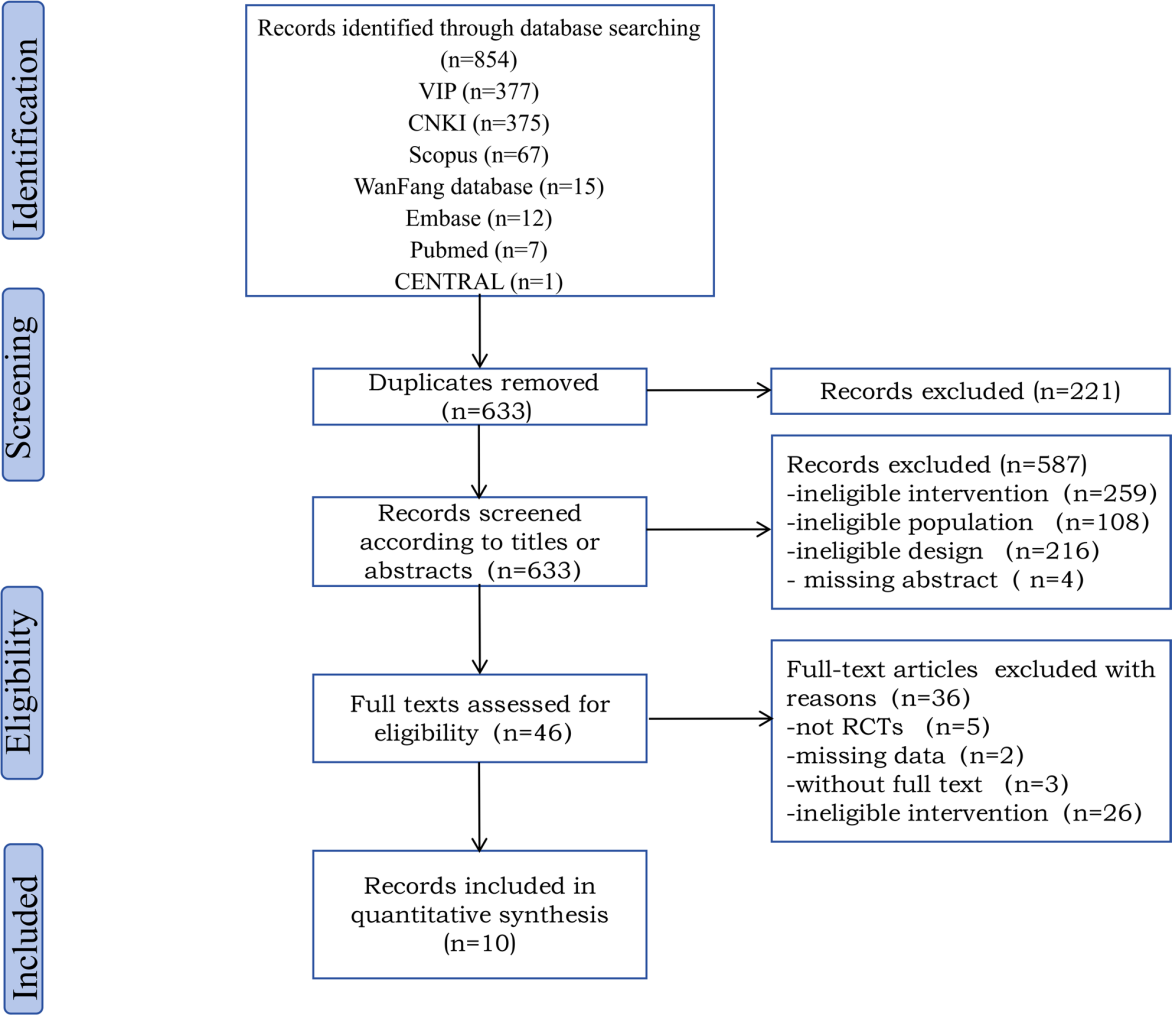
Flow diagram for study inclusion and exclusion

The research results were exported to Endnote 20.0. The duplicates were identified and removed automatically. Two reviewers (S.Y.Z and X.L.Z) independently examined all titles and abstracts, followed by a full-text review by the same two reviewers. Any discrepancies were settled through a full examination and discussion. If necessary, a third reviewer (Z.Z.W) was invited to reach a consensus. The following information was extracted from each study: (i) characteristics of the study (i.e., authors, city, year of publication); (ii) characteristics of the population (i.e., sample size, age); (iii) characteristics of the intervention (i.e., treatment setting, duration, injection dose, number of injections and points); (iv) characteristics of the outcomes (i.e., means and standard deviations of the above indicators). If the extracted data were not reported in the article, we emailed the first/corresponding author of that article to secure the missing data (Fig. 1).

### Quality assessment and strength of recommendations

S.Y.Z and C.L., two independent reviewers, assessed the quality of the eligible studies, and any differences were reconciled through a thorough examination and discussion until a consensus was reached. The Cochrane risk-of-bias tool for randomized trials encompasses seven domains [34]: (i) Random sequence generation (selection bias); (ii) Allocation concealment (selection bias); (iii) Blinding of participants and personnel (performance bias); (iv) Blinding of outcome assessment (detection bias); (v) Incomplete outcome data (attrition bias); (vi) Selective reporting (reporting bias); (vii) Other bias. Each study's risk of bias was classified as "low risk," "unclear risks," or "high risk". Studies were classified as high-risk if ≥ 2 key domains (randomization, allocation concealment, blinding, incomplete data and selective reporting) showed high bias risk, ensuring maximize the reliability of our sensitivity analyses. The Strength of Recommendation Taxonomy (SORT) was employed to classify each study into three levels of evidence and recommendation, ranging from 1 to 3 [35]. Level 1 was based on good-quality patient-oriented evidence, Level 2 was based on limited-quality patient-oriented evidence, and Level 3 was based on other evidence. The three strengths of recommendation were A to C, with Recommendation A: Based on consistent and good-quality patient-oriented evidence; Recommendation B: Based on evidence of varying or inadequate patient-oriented quality; Recommendation C: A recommendation based on consensus, customary practice, opinion, disease-related data, or case studies for diagnosis, treatment, prevention, or screening. Low-quality (Recommendation C) studies were excluded.

### Statistical analysis

A random-effects model was employed by RevMan 5. 4. 1 to assess the effect of ultrasound-guided BoNT-A injection interventions on the alleviation of pain in patients, with standardized mean differences (SMD) and 95% confidence intervals (CI) [36]. The SMD was classified as either small (from 0.2 to 0.49), moderate (from 0.50 to 0.79), or large (equal to or above 0.80) [37]. The I^2^ statistic among studies was utilized to assess heterogeneity, with the I^2^ index level being either low, medium, or high when the level was > 25%, > 50% or > 75%, respectively [38]. In addition, a sensitivity analysis was performed by removing them on a case-by-case basis. Publication bias was assessed through visual inspection of funnel plots, with asymmetry suggesting potential bias. Egger's linear regression test was performed to statistically evaluate funnel plot asymmetry [39], and the trim-and-fill method was applied to adjust for potential publication bias when significant asymmetry is detected [40]. *P* < 0.05 was considered to indicate statistical significance.

## Results

### Study selection

Out of the 854 articles initially obtained through the database, 221 were eliminated due to duplication, and 587 were excluded after the titles and abstracts read. Ultimately, only 10 of the 46 eligible to read the full text were included. The other 36 studies were excluded for the following reasons: not RCTs (*n* = 5), missing data (*n* = 2), missing full text (*n* = 3), and ineligible interventions (*n* = 26). The qualitative analysis was satisfactory for a total of 10 articles (Fig. 1).

### Characteristics of the included studies

Our systematic analysis incorporated data from 533 participants across 10 randomized controlled trials (RCTs) published between 2017 and 2022, including nine studies published in Chinese [41–49] and one in English [50]. The included studies demonstrated variable interventions: one trial utilized BoNT-A alone [44], four incorporated BoNT-A with rehabilitation interventions [41, 45, 49, 50], and five combined BoNT-A with pharmacological adjuvants. Of these five studies, lidocaine in two studies [43, 47], lidocaine with movement therapy in two studies [42, 46], and triamcinolone acetonide plus rehabilitation in one trial [48].

The majority of studies utilized single-injection [41, 42, 44–50], contrasting with one trial that employed a two-dose administration schedule [43]. For the total injectable dose, one study injected 200 U [43], another study injected 200–400 U [49], and eight studies injected 100 U [41, 42, 44–48, 50]. The follow-up periods spanned from 4 to 12 weeks for all studies. Regarding outcome measures, ten indicators were included in the eligible studies. The VAS score was commonly used in nine studies [41, 43, 45–50], the UEFMA score was eight [41, 43, 45–50] and the MBI score was two [48, 49]. Shoulder mobility was assessed across three movement dimensions: five studies measured full shoulder mobility (flexion, abduction, external rotation) [42, 43, 46–48], compared to three studies that assessed only two dimensions (flexion/abduction [45] or abduction/external rotation [49, 50]). The MAS score and QoL score were measured in the study by Tan et al. [50]. Similarly, the Brief McGill Pain score and Clinical Efficacy Assessment were only applied in the article by Wang et al. [44] (Tables 1 and 2).

**Table 1:** The characteristics of the included studies

Study, Year	City	Patient Characteristics		Group		Number of Injections	Total Injection dose	Duration	Measurement^b^
		N (E/C)^a^	Age (E/C)	Experimental	Control				
Lin et al., 2021	Ningde, Fujian	30/30	63. 14 ±4. 32	BoNT-A& Conventional Rehabilitation	Conventional Rehabilitation	Once	100U	12 weeks	①②
Ma et al., 2017	Shaoguan, Guangdong	25/25/25	67 ± 8.3/ 65 ± 7.8/ 63 ± 8. 9	BoNT-A& Conventional Rehabilitation(A)	Triamcinolone acetonide & Conventional Rehabilitation (B) Conventional Rehabilitation (C)	Once	100U	4 weeks	①②③④
Wei et al., 2022	Nanning, Guangxi	14/16	68.86 ± 9.81/ 54.44 ± 11.7	BoNT-A& Conventional Rehabilitation	Conventional Rehabilitation	Once	200-400U (4 points)	4 weeks	①②④⑤⑩
Zhang et al., 2022	Nanning, Guangxi	25/25	54.12 ± 11.33/ 54.76 ± 10.77	BoNT-A &Triamcinolone &Conventional Rehabilitation	Conventional Rehabilitation	Once	100U (3 points)	4 weeks	①②③④⑤⑩
Tan et al., 2021	Chongqing	18/18	51.1 ± 11.4/ 53.9 ± 13.0	BoNT-A &Conventional Rehabilitation	Saline &Conventional Rehabilitation	Once	100U (2 points)	24 weeks (VAS; the ROM of abduction and external rotation) 4 weeks (UEFMA; flexion)	①②④⑤⑥⑦
Wu et al., 2018	Wenzhou, Zhejiang	20/20	59.53 ± 1.52/ 62.72 ± 2.11	BoNT-A&lidocaine &Conventional Rehabilitation	Conventional Rehabilitation	Once	100U	4 weeks	①③④⑤
Yu et al., 2020	Tianmen, Hubei	40/38	60.88 ± 4.62/ 61.34 ± 4.19	BoNT-A&lidocaine &Conventional Rehabilitation	Triamcinolone &Lidocaine &Conventional Rehabilitation	Once	100U	8 weeks	①②③④⑤
Sun et al., 2022	Xian, Shanxi	54/54	60.12 ± 3.09/ 59.26 ± 3.18	BoNT-A&Lidocaine carbonate	Triamcinolone Acetonide Acetate &Liocaine Carbonate	Once a week, twice in total	200U (100U each time)	8 weeks	①②③④⑤
Wang et al., 2021	Xinchang, Zhejiang	21/21	43.88 ± 11.34	BoNT-A	Lidocaine &Compound Betamethasone	Once	100U (20U each point)	12 weeks	⑧⑨
Zhang et al., 2023	Nanning, Guangxi	25/25	54.44 ± 11.05	BoNT-A &Triamcinolone &Conventional Rehabilitation	Conventional Rehabilitation	Once	100U (3 points)	4 weeks	①②③④⑤⑩
Wang et al., 2017	Qingdao, Shandong	19/20	59.5 ± 9.5/ 62.7 ± 11	BoNT-A&Lidocaine	Triamcinonide& Lidocaine	Once	100U	4 weeks	①②③④⑤

^*a*^E = experimental group; C = control group

^*b*^①VAS score; ②FMA score; ③Shoulder Flexion ROM; ④Shoulder Abduction ROM; ⑤Shoulder External Rotation ROM; ⑥MAS score; ⑦Qol score; ⑧Simple McGill pain score; ⑨Clinical efficacy evaluation; ⑩MBI

**Table 2:** The results of the included studies

Study, Year	Results							
	Effectiveness	VAS score (E/C)	FMA score (E/C)	Flexion ROM (E/C)	Abduction ROM (E/C)	External Rotation ROM (E/C)	MBI score (E/C)	Other outcomes (E/C)
Lin et al., 2021	Ultrasound > Control	4.01 ± 1.38/ 4.14 ± 1.12	12.0 ± 4.4/ 11.6 ± 4.0					
Ma et al., 2017	Ultrasound > Control	2.34 ± 1.10/ 5.40 ± 0.50	49. 3 ± 7.4/ 35.8 ± 7.9	103.7 ± 11.2/ 54.8 ± 7.6	91.5 ± 7.4/ 46.3 ± 2.4			
Wei et al., 2022	Ultrasound > Control	2.07 ± 0.62/ 3.44 ± 1.50	22.64 ± 4.94/ 24.81 ± 5.23		83.57 ± 6.33/ 78.86 ± 4.79 Not statistically significant	47.74 ± 1.86/ 37.29 ± 1.74	62.86 ± 15.29/ 49.36 ± 19.14	
Tan et al., 2021	Ultrasound > Control	4.22 ± 1.70/ 5.17 ± 1.34	29.67 ± 12.46/ 23.94 ± 10.06 Not statistically significant		76.50 ± 23.24/ 70.83 ± 26.30	39.50 ± 11.63/ 33.93 ± 10.44		-MAS external rotation 2.39 ± 0.50/ 2.72 ± 0.57 abduction 2.44 ± 0.62/ 2.56 ± 0.98 -QoL P = 0.025
Wu et al., 2018	Ultrasound > Control	5.45 ± 1.83/ 7.80 ± 1.43		93.42 ± 14.45/89.23 ± 7.67	80.48 ± 7.35/ 76.92 ± 9.47 Not statistically significant	33.82 ± 9.84/ 27.48 ± 12.39 Not statistically significant		
Yu et al., 2020	Ultrasound > Control	7.81 ± 1.04/ 5.52 ± 0.86	35.12 ± 3.68/ 45.72 ± 4.87	87.93 ± 6.38/ 93.58 ± 7.19	80.43 ± 6.49/ 80.51 ± 6.63	28.16 ± 6.59/ 34.96 ± 8.56		
Sun et al., 2022					Not statistically significant			
Wang et al., 2021	Ultrasound > Control							-McGill score 3.43 ± 2.16/17.00 ± 7.96 - Clinical efficacy 61.91/ 33.34
Zhang et al., 2022	Ultrasound > Control	4.44 ± 0.917/ 5.92 ± 0.759	45.6 ± 2.739/ 25.32 ± 2.824	130.8 ± 8.50/ 106.48 ± 8.564	135.80 ± 8.50/ 111.48 ± 8.733	35.28 ± 4.766/ 20. 68 ± 2.982		
Wang et al., 2017	Ultrasound > Control	5.4 ± 1.8/ 7.8 ± 1.4	11.9 ± 4.8/11.5 ± 5.1	93.2 ± 14.5/ 85.2 ± 7.8	80.5 ± 7.1/ 76.8 ± 9.7 Not statistically significant	33.2 ± 9.7/ 27.1 ± 12.5		

^*a*^E = experimental group; C = control group

### Assessment of bias

**Fig. 2:**
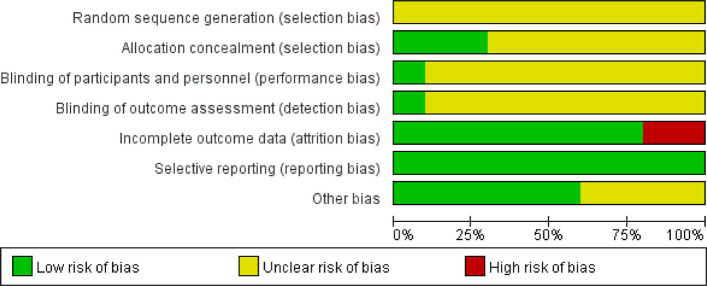
Evidence synthesis risk of bias summary of included trials. ^a^The colors in the bars represent different categories: red = high bias, green = low bias, yellow = unclear bias

**Fig. 3:**
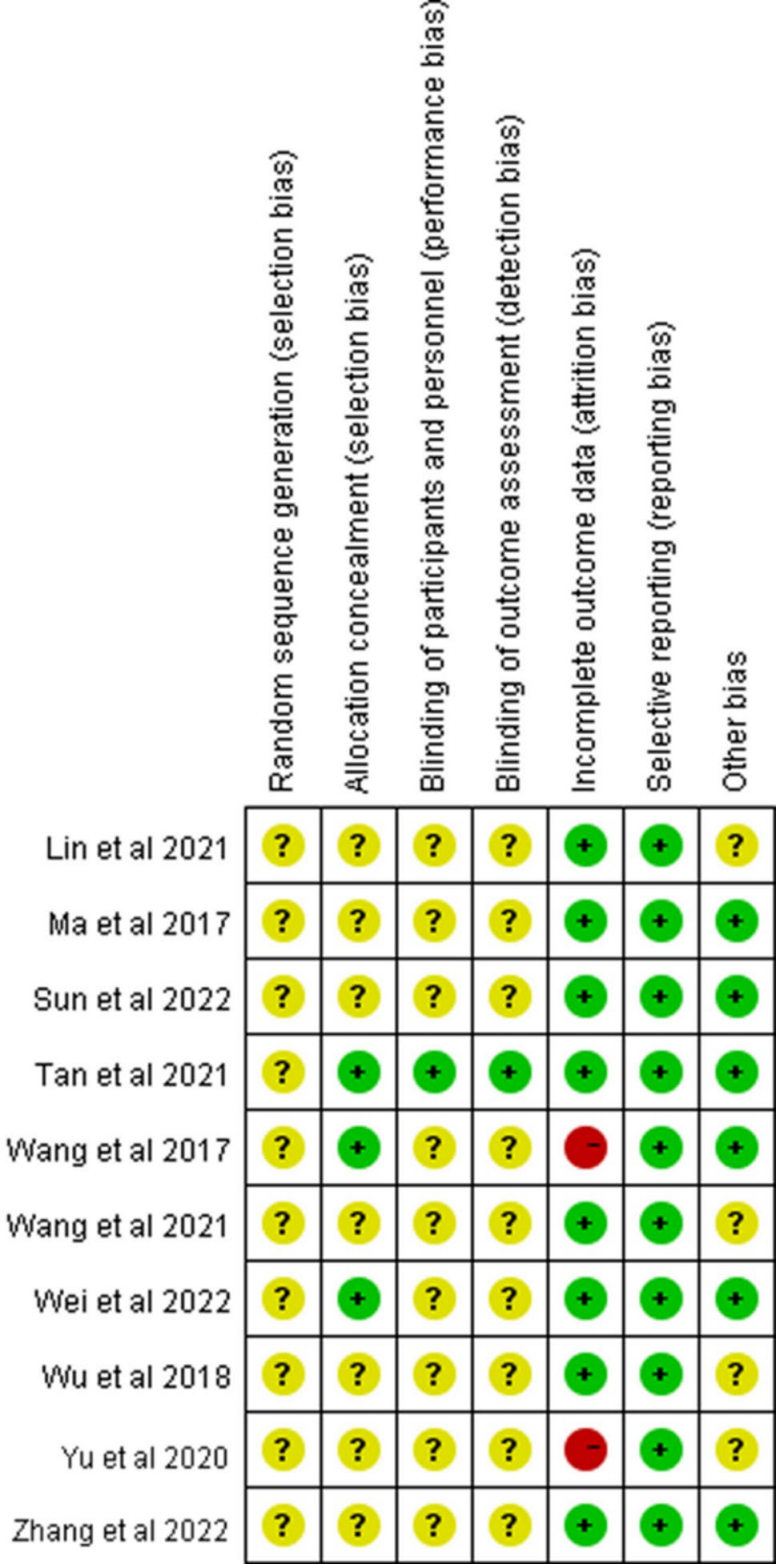
Risk of bias assessment for included studies

The overall risk of bias across the 10 studies raised important methodological concerns that warranted careful interpretation. While seven studies adequately described randomization using random number tables [41, 43–45, 47–49], three failed to report specific methods [42, 46, 50]. The blinding procedures were particularly poorly documented, with only one trial clearly describing double-blinding methods [50]. Although two studies had attrition issues [46, 47], the result showed that these did not substantially alter the primary conclusions (I^2^ changed from 71 to 73%). While eight trials were graded as level A evidence, the unclear randomization (30% of studies) and vague blinding reporting (90%) suggested that the pooled estimates may represent upper-bound treatment effects, particularly for subjective outcomes (Fig. 2, Fig. 3, Supplementary file 2: Table S1).

### VAS score

**Fig. 4:**
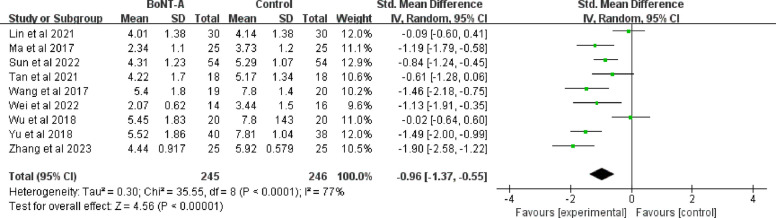
Forest plot of VAS pain score

As shown in Fig. 4, nine RCTs analyzed the VAS score, including a total of 491 participants (245 in the BoNT-A group and 246 in the control group) [41–43, 45–50]. A pooled mean standard deviation difference of −1.1 (95% CI −1.47 to −0.73;* P* < 0.001) was revealed to favor of the BoNT-A group. There was a high heterogeneity across the studies (I^2^ = 77%; *P* = 0.0001), which may be partly explained by the subjective self-rating of pain measured from VAS.

**Fig. 5:**
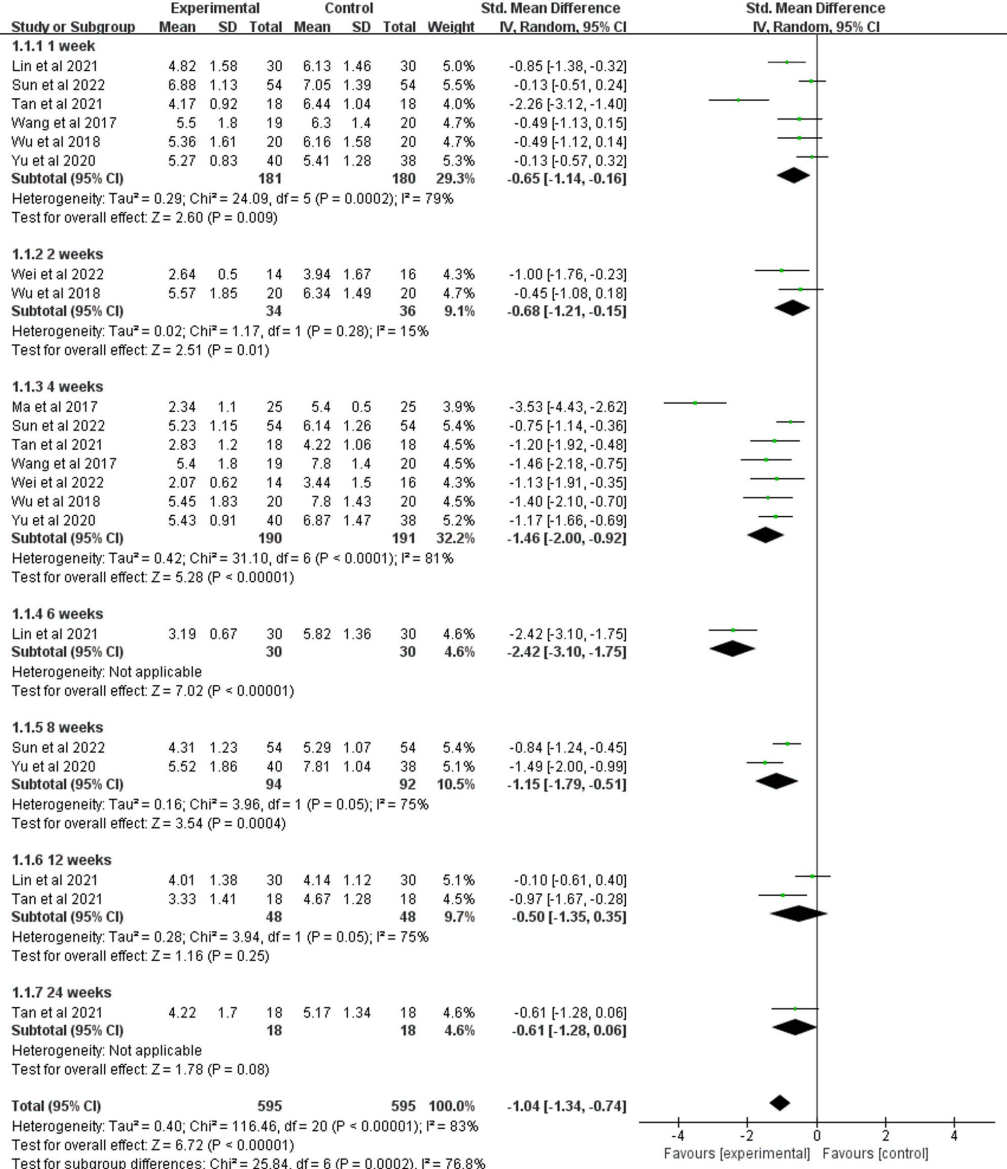
Results of BoNT- A on VAS score in different follow-up periods

To analyze heterogeneity, subgroup analyses were performed according to the follow-up periods, baseline pain level, sex ratio, and stroke type. Of the 9 studies, 6 reported VAS scores at one-week post-injection [41, 43, 45–47, 50], 2 reported scores at 2 weeks [47, 49], 7 reported scores at 4 weeks [42, 43, 45–47, 49, 50], 2 reported scores at 8 weeks [43, 46], and 2 reported scores at 12 weeks [41, 50]. The results showed that pain relief was most pronounced at 4 weeks (SMD = −1.46; 95% CI −2 to −0.92; *P* < 0.001). Minimal improvement was observed at 12 weeks [41, 50], although this difference was not statistically significant (SMD = −0.5; 95% CI −1.34 to 0.35; *P* = 0.05). Overall, there were significant differences in outcomes at 1, 2, 4, 6, 8, 12, and 24 weeks of follow-up (*P* < 0.0001). The subgroup analysis showed that baseline pain score was the main source of efficacy heterogeneity (I^2^ decreased from 71% to 35.6%), while gender and stroke type had relatively limited effects (I^2^ = 42.9% and 0%, respectively). Although the final heterogeneity by stroke type group was 0%, there was still significant heterogeneity within the ischemic stroke group (I^2^ = 85%) (Fig. 5, Supplementary file 3: Fig. S1-S3).

### UEFMA score

**Fig. 6:**
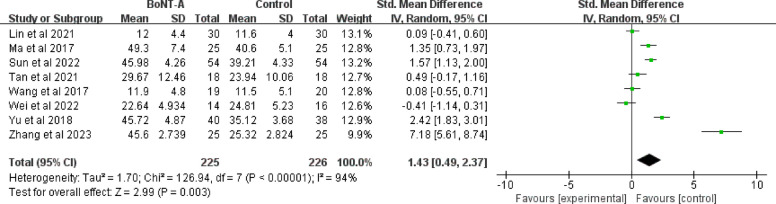
Forest plot of UEFMA score

The UEFMA score was reported in eight studies with a total sample size of 451 (225 in the BoNT-A group and 226 in the control group). For analysis, a random-effect model was employed due to the considerable heterogeneity (I^2^ = 94%, *P* = 0.003). The use of different injection routes may be a factor contributing to the high heterogeneity. Four studies used intra-articular injections [42, 43, 45, 46], two used intramuscular injections [49, 50], and two used injections into the tendon sheaths and bursa [45, 48]. The pooled results revealed a significant improvement of BoNT-A on upper limb functional recovery (SMD = 1.43; 95% CI 0.49 to 2.37; *P* = 0.003) (Fig. 6).

To explore the sources of heterogeneity, we performed subgroup analysis based on different injection routes, age and intervention. The results showed that there was no significant difference among different injection routes (*P* = 0.17). Intra-articular injections showed a non-significant trend toward benefit (SMD = 1.04; 95% CI −0.03 to 2.12; *P* = 0.06) with extreme heterogeneity (I^2^ = 94%), where two studies reported large effects (Sun 2022: SMD = 1.57; Yu 2020: SMD = 2.43). Similarly, intramuscular approaches demonstrated no significant advantage (SMD = 0.06; 95% CI −0.83 to 0.95; *P* = 0.9) with moderate heterogeneity (I^2^ = 68%). The bursa/tendon subgroup showed the greatest improvement (SMD = 4.22) but was impaired by extreme outliers (Zhang 2022: SMD = 7.18), showing non-significant results (*P* = 0.15) and almost complete heterogeneity (I^2^ = 98%). There was high heterogeneity within age groups (I^2^ = 97% for the group < 60 years and I^2^ = 93% for the group ≥ 60 years), but the between-group heterogeneity was 0%, indicating that age was not a major contributor to variation in treatment effect. The high heterogeneity among the different interventions (I^2^ = 82.8%) suggested that their difference was statistically significant (Supplementary file 3: Fig. S4-S6).

## ROM of the shoulder

### The ROM of shoulder flexion

**Fig. 7:**
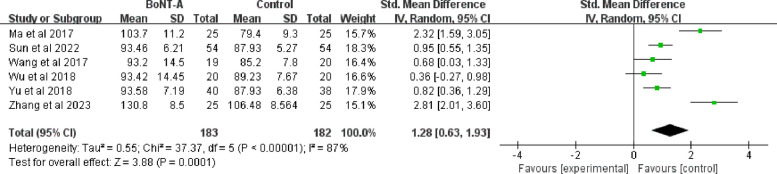
Forest plot of the ROM of shoulder flexion

A total sample size of 365 (183 in the BoNT-A group and 182 in the control group) was reported in six studies [42, 43, 45–48], revealing a significant heterogeneity among them (I^2^ = 87%; *P* < 0.001) in the ROM of shoulder flexion. The pooled analysis of these six trials showed that a larger flexion angle was associated with the BoNT-A group than the control group (SMD = 1.28; 95%CI 0.63 to 1.93; *P* < 0.001) (Fig. 7).

**Fig. 8:**
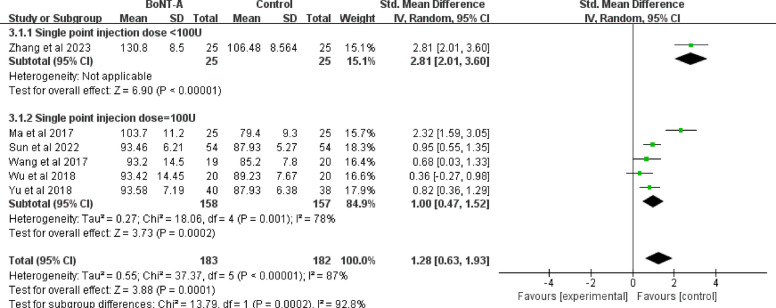
Results of BoNT- A on ROM of flexion with different injection doses for singe point

Subgroup analyses were performed according to different injection doses, geographic locations, and control types to explore the sources of heterogeneity. Of the six included studies, only one had a single-site injection dose of less than 100 U [48]. According to the subgroup meta-analysis, the ROM of shoulder abduction differed between subgroup1 (Single -point injection dose < 100 U) and subgroup 2 (Single point injection dose = 100 U) (*P* = 0.0002). Geographic stratification substantially reduced overall heterogeneity (I^2^ decreased from 87% to 26.5%), indicating that regional variations partially accounted for treatment effect differences, although significant heterogeneity persisted within Southern China (I^2^ = 91%). The control-type analysis revealed superior treatment effects for conventional rehabilitation alone (SMD = 1.81; 95% CI 0.28 to 3.35) compared to conventional rehabilitation combined with adjunctive drug injections (SMD = 0.86; 95% CI 0.58 to 1.93), suggesting that this distinction may represent another source of effect variability (I^2^ decreased from 87% to 30.4%) (Fig. 8, Supplementary file 3: Fig. S7-S8).

### The ROM of shoulder abduction

**Fig. 9:**
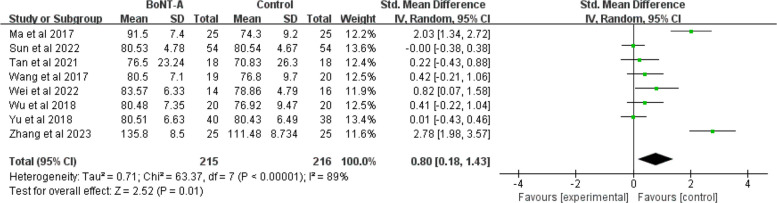
Forest plot of the ROM of shoulder abduction

A total sample size of 431 (215 in the BoNT-A group and 216 in the control group) was reported in eight studies [42, 43, 45–50]. The heterogeneity with 89% of shoulder abduction ROM was high (*P* < 0.001). The pooled analysis of these eight trials revealed that a higher abduction angle was associated than BoNT-A group compared to the control group (SMD = 0.8; 95%CI 0.18 to 1.43; *P* = 0.01) (Fig. 9).

Subgroup analysis was performed according to different sample numbers, injection points, and dilution concentrations to explore the source of heterogeneity. The sample size of the experimental group varied for each study, with a maximum of 54 and a minimum of 14. Two studies had an experimental group sample size of more than 30 people [43, 46] and six had no more than 30 people [42, 45, 47–50]. In the subgroup meta-analysis, the ROM of shoulder abduction was different between subgroup 1 (experimental group ≤ 30 people) and subgroup 2 (experimental group > 30 people) (*P* = 0.01). While the number of injection sites did not account for outcome variability, dilution concentration may represent a potential source of heterogeneity (I^2^ = 0% and 88.5%, respectively). The consistently high within-group heterogeneity across all analyses suggested that other unmeasured technical or clinical factors likely influenced treatment outcomes (Supplementary file 3: Fig. S9-S11).

### The ROM of external rotation

**Fig. 10:**
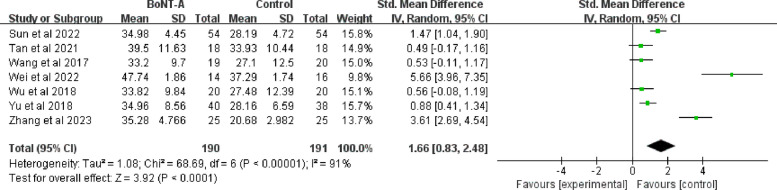
Forest plot of the ROM of shoulder external rotation

There were seven trials with 381 patients who compared the control group to the BoNT-A group in terms of external rotation (190 in the BoNT-A group and 191 in the control group) [42, 43, 46–50]. Although the result is conducive to BoNT-A group in improvement of shoulder external rotation mobility (SMD = 1.66; 95%CI 0.83 to 2.48; *P* < 0.001), a high heterogeneity was observed in the pooled analysis (I^2^ = 91%; *P* < 0.001) (Fig. 10).

To explore the sources of heterogeneity, the seven studies were grouped according to study quality assessment, duration of disease and follow-up. Two articles were classified as Grade B because some of participants dropped out [46, 47]. Specifically, the study from Yu et al. lost 6 cases [46] and the study from Wang et al. lost 9 cases [47]. A significant difference was observed between the Grade A subgroup and Grade B subgroup (*P* = 0.01). Although subgroup analysis of disease duration reduced the final heterogeneity to 0%, it remained extremely heterogeneous (I^2^ > 90%) within groups, suggesting that disease duration was not the true source of variation. Subgroup analysis of follow-up time showed that the highest heterogeneity at 2 weeks (I^2^ = 97%), and the difference between groups was significant (P = 0.008), indicating that evaluation time was the key factor affecting the consistency of results. (Supplementary file 3: Fig. S12-S14).

### MBI score

Two trials with 80 patients that compared the BoNT-A group to the control group [48, 49]. There was a notable heterogeneity among the BoNT-A and the control group (39 and 41 respectively) (I^2^ = 79%; *P* = 0.03). However, subgroup analyses cannot be applied to explore sources of heterogeneity because of the limited number of studies. The results indicated that the MBI score of the BoNT-A group was greater than the control group (SMD = 1.33; 95% CI 0.22 to 2.43; *P* = 0.02) (Supplementary file 2: Fig. S15).

### Other outcomes

One record reported the QoL scale score and MAS score [50]. Another study reported simple McGill pain score and clinical efficacy [44]. Because of the lack of studies on the four indexes mentioned above, this meta-analysis was unable to create forest plots for them.

### Sensitivity analysis

The sensitivity analyses were performed by removing each trial and assessing how it affected the primary outcomes. No significant differences were found for the UEFMA score and ROM of shoulder, confirming that these results are robust. The heterogeneity of the VAS score was significantly reduced to 44% following the exclusion of Lin et al. involving patients with obviously shorter disease duration (< 2 months) [41], suggesting that disease duration may represent a significant source of clinical heterogeneity. The variation likely reflects stroke recovery stages: BoNT-A demonstrates superior therapeutic efficacy in chronic spasticity management, where well-established neuromuscular dysfunction and structural adaptations are present, compared to early-stage recovery when spasticity patterns remain incompletely formed. The remaining studies demonstrated consistent treatment effects (SMD = −1.23; 95% CI −1.52 to −0.94), supporting the robustness of our primary findings. Flexion and abduction ROM was not statistically significant following exclusion of Zhang et al., possibly attributable to the three injection routes [48].

### Publication bias

Funnel plot symmetry was observed for VAS and UEFMA outcomes, with Egger's tests showing no significant publication bias (VAS: *P* = 0.273; UEFMA: *P* = 0.323). Quantitative assessment of publication bias was not performed for MBI, QoL scale score, MAS score, simple McGill pain score, and clinical efficacy due to insufficient studies. While visual asymmetry was noted in funnel plots for shoulder flexion and external rotation, Egger's tests did not reach statistical significance (flexion: *P* = 0.232; external rotation: *P* = 0.133), possibly reflecting limited study numbers or heterogeneity among smaller studies. Significant funnel plot asymmetry was detected for abduction measurements (Egger's test *P* = 0.033). Following trim-and-fill adjustment, the effect estimate remained statistically significant (SMD = 0.817; 95% CI 0.183 to 1.452; *P* = 0.012), supporting the therapeutic benefit of ultrasound-guided BoNT-A for improving abduction ROM (Supplementary file 3: Fig. S16-S18).

## Discussion

The application of botulinum toxin type A (BoNT-A) injections has become increasingly widespread in recent years, with demonstrated efficacy in treating diseases such as strabismus [51], cerebral palsy [52] and sialorrhea [53]*.* Substantial evidence supported that BoNT-A is a good choice for pain relief [16–18], because of its ability to inhibit of acetylcholine release and other neurological factors. The purpose of this meta-analysis was to investigate the efficacy of ultrasound-guided injection of BoNT-A for treating shoulder pain. Our analysis of 10 studies (9 hemiplegic shoulder pain [41–43, 45–50], 1 myofascial pain [44]) demonstrated significant pain reduction and functional improvement. Sensitivity analyses following exclusion of Lin et al. [41] (reducing VAS score heterogeneity from 71 to 44%) confirmed the robustness of primary outcomes. However, significant publication bias for abduction (*P* = 0.033) and nonsignificant funnel plot asymmetries for flexion and external rotation (both *P* > 0.05) necessitate cautious interpretation of effect estimates.

The results showed that BoNT-A under ultrasound guidance can reduce shoulder pain greatly in the nine included studies, with a reduction of 1.1. In this meta-analysis, the results at 1, 2, 4,6, 8, 12 and 24 weeks of follow-up were analyzed. Current evidence suggested that the therapeutic effect of BoNT-A may peak around 4 weeks after injection, though this time point should be interpreted cautiously, as only two included studies provided follow-up data at 12 weeks. Consistent with our findings, several studies identified peak efficacy at 4 weeks [43, 46], whereas others reported maximal therapeutic effects at 12 weeks [54, 55]. The limitation of BoNT-A's analgesic effects highlights the potential value of combination therapies during this optimal treatment window.

For upper limb function, the meta-analysis showed that the ultrasound-guided BoNT-A group was superior to control group in terms of improvement in the eight studies (SMD = 1.43; 95% CI 0.49 to 2.37; *P* = 0.003). There was a divergence between subgroup trends and statistical significance: while combined tendon-bursa injections made the greatest improvement for efficacy, no individual subgroup reached significance. The intra-articular route demonstrated promising results, likely reflecting differences in underlying pathology (spasticity versus joint pain). The non-significant benefit observed with intramuscular injections may indicate that isolated muscle targeting is insufficient for functional improvement in the stroke population. The synergistic effects of BoNT-A through intra-articular diffusion and muscle tone modulation may explain the superior outcomes observed with combined approaches, particularly for patients with mixed spasticity-pain presentations [56] These findings emphasize the need for individualized injection strategies based on each patient's predominant pathophysiology.

Our analysis demonstrated significant improvements in three directions of shoulder following ultrasound-guided BoNT-A injections, with external rotation showing the greatest benefit (SMD = 1.66) compared to abduction (SMD = 0.8). These findings contrasted with the previous report from Xie et al. [54], which suggested that BoNT-A treatment did not improve flexion ROM. Potential explanations for this discrepancy include variations in both study population characteristics and injection techniques. Notably, while our analysis predominantly included subacute cases (≤ 6 months duration in 80% of studies), the cohort examined by Xie et al. consisted mainly of chronic patients (> 6 months in 56% of cases), whose fibrotic changes may compromise treatment responsiveness [57]. Technical factors also influenced outcomes, six studies included in this article used ultrasound-guided injections but Xie et al. adopted ultrasound or electromyography guidance. Split-point injections (< 100U/site) proved more effective for improving flexion than single-point injections, possibly due to broader medication distribution [58]. While subgroup analyses revealed greater abduction improvements in older patients and smaller cohorts, these observations require cautious interpretation given sample size limitations. Two studies showed modest attrition rates (7.14–15.2%) [46, 47], though below the 20% threshold that typically raises concern for outcome bias [59]. These results collectively highlight the importance of patient selection, injection technique, and dosing strategy in optimizing BoNT-A outcomes for shoulder mobility.

In terms of the MBI score, the results showed that BoNT-A facilitated the improvement of MBI score under ultrasound guidance. Shoulder pain, disability, and depression affect stress, then stress further affects quality of life [60]. Therefore, clinical treatment should be carried out from both physical and psychological perspective to improve the quality of life. Moreover, age may be a key factor in regulating quality of life. Disability increases with age, although older patients have better mental health than younger patients [61].

While our pre-specified subgroup analyses examined duration of follow-up, route of injection, single point dose, sample size, and study quality, other important sources of clinical heterogeneity warrant further discussion. Patient-level variations, including the wide age range (39–78 years), broad disease duration (1.35–15.08 months), differing baseline pain severity (VAS 5.38–9.7), and stroke types (infarction vs. hemorrhage), may account for differential treatment responses.

Intervention-related differences likely represent another key factor, particularly regarding injection protocols (total dosage 100-400U, 1–4 injection points, dilution concentrations 50-100U/ml) and comparator types (conventional rehabilitation, placebo, or other injectable medications). Methodological variations across studies further contributed to the observed heterogeneity, especially in assessment methods (unclear specification of ROM measurements in 3 studies) and risk of bias. Evidence suggests that ambiguous randomization reporting and unblinded study designs may increase the treatment effect by approximately 10% and 68%, respectively [62, 63]. These limitations emphasize the need for cautious interpretation of the results, with particular weight given to findings from the double-blinded trial [50].

The strength of this study is that, to the best of our knowledge, this is the first retrospective meta-analysis of ultrasound-guided BoNT-A injections for shoulder pain. The current study provides evidence for the pain relief effect of ultrasound-guided BoNT-A on the shoulder. Next, all 10 studies included in this analysis used an RCT design, making the results relatively reliable. Finally, a sensitivity analysis was conducted by excluding each one and all results were found to be robust. The robust results further strengthen the credibility of this analysis. Several important limitations of this study should be acknowledged. Firstly, the evidence base is limited by the small scale of most studies, with only 2 of the 10 included trials enrolling more than 30 patients [43, 46]. Secondly, we observed considerable statistical heterogeneity across most outcome measures despite conducting pre-specified subgroup analyses. Finally, the interpretation of abduction outcomes requires caution due to detected publication bias. The reliability of flexion and external rotation results remains uncertain given their visual funnel plot asymmetry and nonsignificant Egger's tests (both *P* > 0.05), potentially reflecting limited statistical power or heterogeneity. To mitigate publication bias, future studies should prioritize prospective registration and larger RCTs with standardized outcomes, which would further strengthen evidence.

## Conclusion

In conclusion, while this systematic review suggests potential benefits of ultrasound-guided BoNT-A injections for improving post-stroke shoulder pain, function, and quality of life, these findings should be interpreted with caution due to substantial heterogeneity, potential publication bias in included studies. In the future, more high-quality studies with standardized protocols, long-term follow-up, and robust blinding procedures are needed to strength evidences for ultrasound-guided BoNT-A in shoulder pain.

## Supplementary Information

Supplementary Material 1. Supplementary file S1: Search formulas for the seven databases listed above.

Supplementary Material 2. Supplementary file S2: Table S1. Study design and quality assessment of randomized studies.

Supplementary Material 3. Supplementary file S3: Figure S1. Results of BoNT- A on VAS score in different baseline pain scores.

Supplementary Material 4. Figure S2: Results of BoNT- A on VAS score in different sex ratios.

Supplementary Material 5. Figure S3: Results of BoNT- A on VAS score in different stroke types.

Supplementary Material 6. Figure S4: Results of BoNT-A on UEFMA score different injection routes.

Supplementary Material 7. Figure S5: Results of BoNT- A on UEFMA score in different age ranges.

Supplementary Material 8. Figure S6: Results of BoNT- A on UEFMA score in different intervention measures.

Supplementary Material 9. Figure S7: Results of BoNT- A on flexion ROM in different geographic locations.

Supplementary Material 10. Figure S8: Results of BoNT- A on flexion ROM in different control types.

Supplementary Material 11. Figure S9: Results of BoNT- A on abduction ROM with different sample sizes of people.

Supplementary Material 12. Figure S10: Results of BoNT- A on abduction ROM in the different numbers of injection.

Supplementary Material 13. Figure S11: Results of BoNT- A on abduction ROM in different dilution concentrations.

Supplementary Material 14. Figure S12: Results of BoNT- A on external rotation ROM in different qualities of articles.

Supplementary Material 15. Figure S13: Results of BoNT- A on external rotation ROM in different durations of disease.

Supplementary Material 16. Figure S14: Results of BoNT- A on external rotation in different follow-up periods.

Supplementary Material 17. Figure S15: Forest plot of the MBI score.

Supplementary Material 18. Figure S16: Funnel plot of all outcomes. a (A) VAS score, (B) UEFMA score, (C) ROM of flexion, (D) ROM of abduction, (E) ROM of external rotation and (F) MBI score.

Supplementary Material 19. Figure S17: Funnel plot with Egger's regression line for abduction ROM.

Supplementary Material 20. Figure S18: Trim-and-fill adjusted plot for abduction ROM

## Data Availability

No datasets were generated or analysed during the current study.

## Abbreviations

BoNT-A: Botulinum Toxin Type A
SSNB: Suprascapular Nerve Block
VAS: Visual Analog Scale
ROM: Range of Motion
UEFMA: Upper Extremity Fugl-Meyer Assessment
BI: Barthel Index
MBI: Modified Barthel Index
MAS: Modified Ashworth Scale
ADL: Activities of Daily Living
QoL: Quality of Life
SMD: Standardized Mean Differences
CI: Confidence Intervals
PRISMA: Preferred Reporting Items for Systematic Reviews and Meta-Analyses
RevMan 5. 4. 1: Review Manager
RCT: Randomized Controlled Trial
SORT: Strength of Recommendation Taxonomy
