# Supplementary material for: Ultrasound-guided botulinum toxin type A for shoulder pain: a meta-analysis of randomized controlled trials
Source: BMC Musculoskelet Disord. 2026 Jan 8;27:14. doi: 10.1186/s12891-025-09347-8 (PMC12781773; doi:10.1186/s12891-025-09347-8)
Supplement: Supplementary file 1 — Supplementary Material 1. Supplementary file S1: Search formulas for the seven databases listed above. [file 12891_2025_9347_MOESM1_ESM.docx]

Pubmed
((((((("Ultrasonography"[Mesh]) OR (Ultrasound[Title/Abstract])) OR (Ultrasounds[Title/Abstract])) OR (Ultrasonic[Title/Abstract])) OR (US[Title/Abstract])) AND (((((((((("Botulinum Toxins, Type A"[Mesh]) OR (Botulinum Toxin A[Title/Abstract])) OR (Toxin A, Botulinum[Title/Abstract])) OR (Botulinum Neurotoxin A)) OR (Neurotoxin A, Botulinum[Title/Abstract])) OR (Botulinum A Toxin[Title/Abstract])) OR (BTXA[Title/Abstract])) OR (BoNT serotype A[Title/Abstract])) OR (BoNT-A[Title/Abstract])) OR (Incobotulinum Toxin A[Title/Abstract]))) AND ((((("Shoulder"[Mesh]) OR (shoulder joint[Title/Abstract])) OR (upper limb[Title/Abstract])) OR (upper extremit*[Title/Abstract])) OR (arm[Title/Abstract]))) AND (("Pain"[Mesh]) OR (((((((Painful[Title/Abstract]) OR (Pains[Title/Abstract])) OR (Suffering, Physical[Title/Abstract])) OR (Physical Suffering[Title/Abstract])) OR (Physical Sufferings[Title/Abstract])) OR (Ache[Title/Abstract])) OR (Aches[Title/Abstract])))

Embase

#1： 'phonophoresis' OR 'radiation, ultrasonic' OR 'sonication' OR 'sonification' OR 'ultra sound' OR 'ultrashell' OR 'ultrasonic' OR 'ultrasonic energy' OR 'ultrasonic irradiation' OR 'ultrasonic measurement' OR 'ultrasonic sound' OR 'ultrasonic wave' OR 'ultrasonic waves' OR 'ultrasonics' OR 'ultrasound radiation' OR 'ultrasound'

#2： 'abobotulinum toxin A' OR 'abobotulinumtoxin A' OR 'abobotulinumtoxinA' OR 'agn 151607' OR 'agn151607' OR 'alluzience' OR 'ant 1207' OR 'ant 1401' OR 'ant 1403' OR 'ant1207' OR 'ant1401' OR 'ant1403' OR 'azzalure' OR 'bocouture' OR 'boe-tox' OR 'BoNT A' OR 'BoNT A DS' OR 'BoNT serotype A' OR 'botox' OR 'botox (100 U) injection' OR 'botox (oculinum)' OR 'botox 100E' OR 'botox a' OR 'botox cosmetic' OR 'botulin A' OR 'botulin toxin a' OR 'botulinium a toxin' OR 'botulinum a exotoxin' OR 'botulinum a toxin' OR 'botulinum neurotoxin a' OR 'botulinum neurotoxin type A' OR 'botulinum toxin type A' OR 'botulinum toxins, type A' OR 'BTXA' OR 'clostridium botulinum a toxin' OR 'clostridium botulinum endotoxin' OR 'Clostridium botulinum neurotoxin A' OR 'Clostridium botulinum neurotoxin type A' OR 'clostridium botulinum toxin type a' OR 'Clostridium botulinum type A neurotoxin' OR 'cnt 52120' OR 'cnt52120' OR 'cunox' OR 'daxibotulinum toxin A' OR 'daxibotulinumtoxin A' OR 'daxibotulinumtoxin A lanm' OR 'daxibotulinumtoxinA' OR 'daxibotulinumtoxinA lanm' OR 'daxibotulinumtoxinA-lanm' OR 'daxxify' OR 'dtx 021' OR 'dtx021' OR 'dwp 450' OR 'dwp450' OR 'dyslor' OR 'dysport' OR 'evabotulinum toxin A' OR 'evabotulinumtoxin A' OR 'evabotulinumtoxinA' OR 'evosyal' OR 'gemibotulinum toxin A' OR 'gemibotulinumtoxin A' OR 'gemibotulinumtoxinA' OR 'gsk 1358820' OR 'gsk1358820' OR 'hu 014' OR 'hu014' OR 'hutox' OR 'incobotulinum toxin A' OR 'incobotulinumtoxin A' OR 'incobotulinumtoxinA' OR 'ipn 10200' OR 'ipn 59011' OR 'ipn10200' OR 'ipn59011' OR 'jeuveau' OR 'letibotulinum toxin A' OR 'letibotulinumtoxin A' OR 'letibotulinumtoxinA' OR 'letybo' OR 'liztox' OR 'lp 09' OR 'lp09' OR 'meditoxin' OR 'mt 10107' OR 'mt 10109' OR 'mt10107' OR 'mt10109' OR 'nabota' OR 'nivobotulinum toxin A' OR 'nivobotulinumtoxin A' OR 'nivobotulinumtoxinA' OR 'nt 201' OR 'nt201' OR 'nuceiva' OR 'oculinum' OR 'onabotulinum toxin A' OR 'onabotulinumtoxin A' OR 'onabotulinumtoxinA' OR 'onaclostox' OR 'pm 12759' OR 'pm12759' OR 'prabotulinum toxin A' OR 'prabotulinumtoxin A' OR 'prabotulinumtoxin A xvfs' OR 'prabotulinumtoxinA' OR 'prabotulinumtoxinA xvfs' OR 'prabotulinumtoxinA-xvfs' OR 'prosigne' OR 'purtox' OR 'qm 1114' OR 'qm1114' OR 'relabotulinum toxin A' OR 'relabotulinumtoxin A' OR 'relabotulinumtoxinA' OR 'reloxin' OR 'rt 002' OR 'rt002' OR 'rtt 150' OR 'rtt150' OR 'vistabel' OR 'vistabex' OR 'xeomeen' OR 'xeomin' OR 'botulinum toxin A'

#3： 'shoulder (body region)' OR 'shoulders' OR 'shoulder'

#4： 'acute pain' OR 'deep pain' OR 'lightning pain' OR 'nocturnal pain' OR 'pain response' OR 'pain syndrome' OR 'treatment related pain' OR 'pain'

#5：#1 AND #2 AND #3 AND #4

Scoupus

"Ultrasonography" OR Ultrasound OR Ultrasounds OR Ultrasonic AND "Botulinum Toxins, Type A"OR Botulinum Toxin A OR Toxin A, Botulinum OR “Botulinum Neurotoxin A”OR “Neurotoxin A, Botulinum”OR “Botulinum A Toxin” OR “BTXA” OR “BoNT serotype A” OR “BoNT-A” OR “Incobotulinum Toxin A” AND "Shoulder"OR “shoulder joint”OR“upper limb” OR “upper extremit*” OR “arm”AND”Pain”OR”Painful”OR”Pains”OR”Suffering,Physical”OR”Physical Suffering”OR”Physical Sufferings”OR”Ache”OR“Aches”

Coherance

#1： Ultrasoud

#2 ：Botulinum Toxins, Type A OR Vistabel Neurotoxin A, Botulinum OR Botulinum Neurotoxin Type A OR Botulinum Toxin A OR Clostridium Botulinum Toxin Type A OR Botulinum A Toxin OR Clostridium botulinum A Toxin OR Toxin, Botulinum A OR Toxin A, Botulinum OR Botulinum Toxin Type A OR Botulinum Neurotoxin A OR Oculinum OR Onabotulinumtoxin A OR OnabotulinumtoxinA OR Meditoxin OR Neuronox OR Vistabex OR Botox

#3： shoulder OR shoulders

#4： Pain OR Pains, Crushing OR Pain, Crushing OR Crushing Pain OR Crushing Pains OR Splitting Pains OR Pain, Splitting OR Splitting Pain OR Pains, Splitting OR Physical Sufferings OR Physical Suffering OR Sufferings, Physical OR Suffering, Physical OR Burning Pain OR Pains, Burning OR Pain, Burning OR Burning Pains OR Ache OR Aches OR Pains, Radiating OR Pain, Radiating OR Radiating Pain OR Radiating Pains OR Pains, Migratory OR Migratory Pains OR Migratory Pain OR Pain, Migratory

#5： Botulinum Toxins, Type A OR Vistabel Neurotoxin A, Botulinum OR Botulinum Neurotoxin Type A OR Botulinum Toxin A OR Clostridium Botulinum Toxin Type A OR Botulinum A Toxin OR Clostridium botulinum A Toxin OR Toxin, Botulinum A OR Toxin A, Botulinum OR Botulinum Toxin Type A OR Botulinum Neurotoxin A OR Oculinum OR Onabotulinumtoxin A OR OnabotulinumtoxinA OR Meditoxin OR Neuronox OR Vistabex OR Botox

#6：#1 AND #2 AND #3 AND #4 AND #5

VIP

“超声” “AND” “肩” “AND” “痛” AND “毒素”

Wanfang

“超声” “AND” “肩” “AND” “痛” AND “毒素”

CNKI

“超声” “AND” “肩” “AND” “痛”
