# Supplementary material for: Ultrasound-guided botulinum toxin type A for shoulder pain: a meta-analysis of randomized controlled trials
Source: BMC Musculoskelet Disord. 2026 Jan 8;27:14. doi: 10.1186/s12891-025-09347-8 (PMC12781773; doi:10.1186/s12891-025-09347-8)
Supplement: Supplementary file 2 — Supplementary Material 2. Supplementary file S2: Table S1. Study design and quality assessment of randomized studies. [file 12891_2025_9347_MOESM2_ESM.pdf]

Table S1. Study design and quality assessment of randomized studies.

Study, Year	design	Random sequence generation	Allocation concealment
Lin et al., 2021	randomized controlled	Yes (random number table method)	Not mentioned
Ma et al., 2017	randomized controlled	Yes (random number table method)	Not mentioned
Wei et al., 2022	randomized controlled	Yes (random number table method）	Not mentioned
Zhang et al., 2022	randomized controlled	Yes (random number table method)	Not mentioned
Tan et al., 2021	randomized controlled	Yes(not described)	Not mentioned
Wu et al., 2018	randomized controlled	Yes(not described)	Not mentioned
Yu. et al., 2020	randomized controlled	Yes(not described)	Not mentioned
Sun et al., 2022	randomized controlled	Yes (random number table method)	Not mentioned
Wang et al., 2021	randomized controlled	Yes (random number table method)	Not mentioned
Wang et al., 2017	randomized controlled	Yes (random number table method)	Not mentioned

Blinding of particiants and personnel	Blinding of outcome assessment	Baseline consistency	Rate of loss to follow-up	Level of evidence
Not mentioned	Not mentioned	Yes	0%	1
Not mentioned	Not mentioned	Yes	0%	1
Not mentioned	Not mentioned	Yes	0%	1
Not mentioned	Not mentioned	Yes	0%	1
Yes	Yes	Yes	0%	1
Not mentioned	Not mentioned	Yes	0%	1
Not mentioned	Not mentioned	Yes	7.14%	2
Not mentioned	Not mentioned	Yes	0%	1
Not mentioned	Not mentioned	Yes	0%	1
Not mentioned	Not mentioned	Yes	15.20%	2
