# Supplementary figures and images for: Ultrasound-guided botulinum toxin type A for shoulder pain: a meta-analysis of randomized controlled trials
Source: BMC Musculoskelet Disord. 2026 Jan 8;27:14. doi: 10.1186/s12891-025-09347-8 (PMC12781773; doi:10.1186/s12891-025-09347-8)

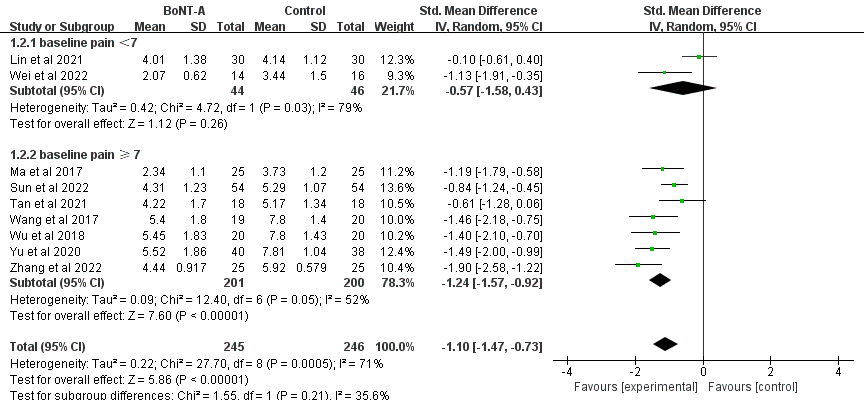

Supplement: Supplementary file 3 — Supplementary Material 3. Supplementary file S3: Figure S1. Results of BoNT- A on VAS score in different baseline pain scores. [file 12891_2025_9347_MOESM3_ESM.png]

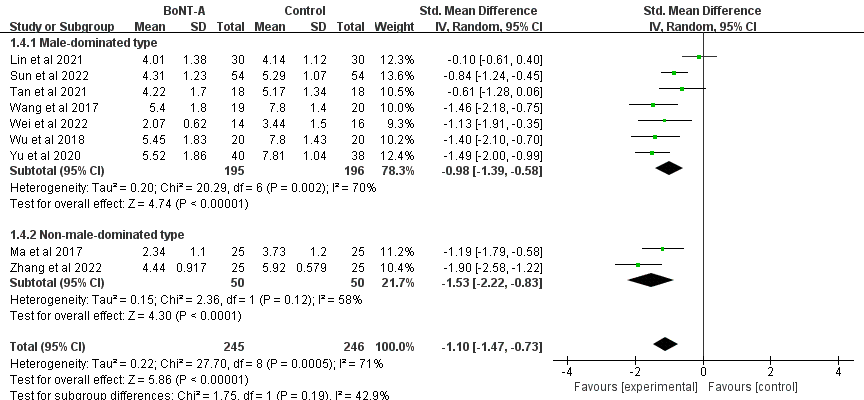

Supplement: Supplementary file 4 — Supplementary Material 4. Figure S2: Results of BoNT- A on VAS score in different sex ratios. [file 12891_2025_9347_MOESM4_ESM.png]

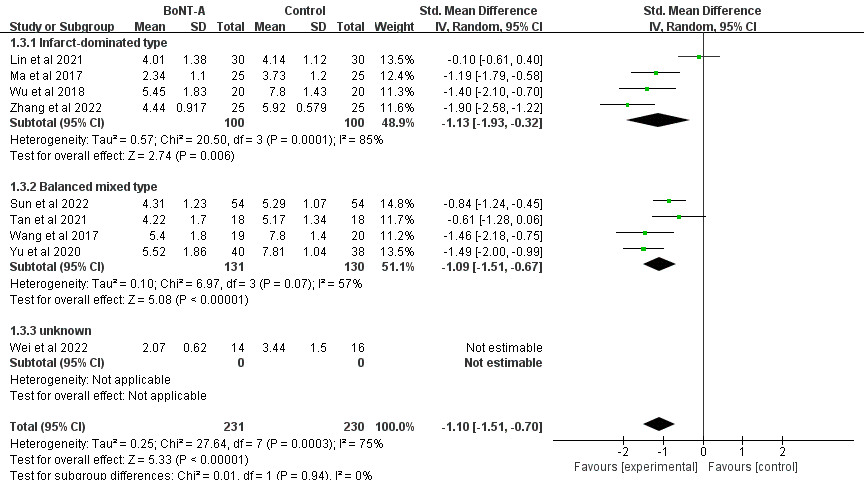

Supplement: Supplementary file 5 — Supplementary Material 5. Figure S3: Results of BoNT- A on VAS score in different stroke types. [file 12891_2025_9347_MOESM5_ESM.png]

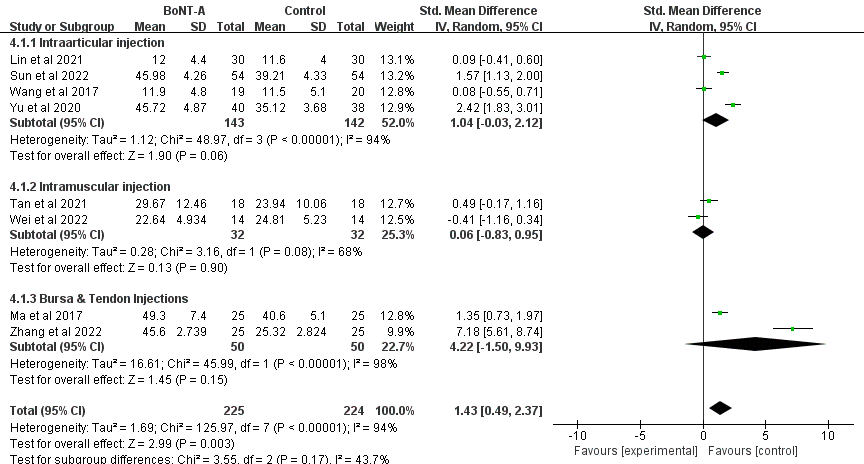

Supplement: Supplementary file 6 — Supplementary Material 6. Figure S4: Results of BoNT-A on UEFMA score different injection routes. [file 12891_2025_9347_MOESM6_ESM.png]

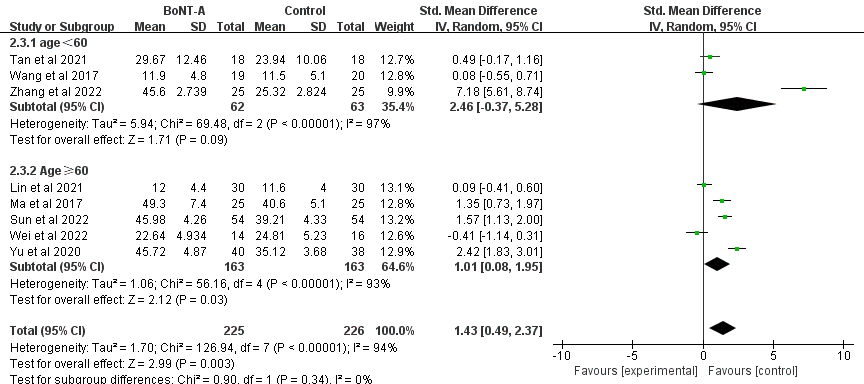

Supplement: Supplementary file 7 — Supplementary Material 7. Figure S5: Results of BoNT- A on UEFMA score in different age ranges. [file 12891_2025_9347_MOESM7_ESM.png]

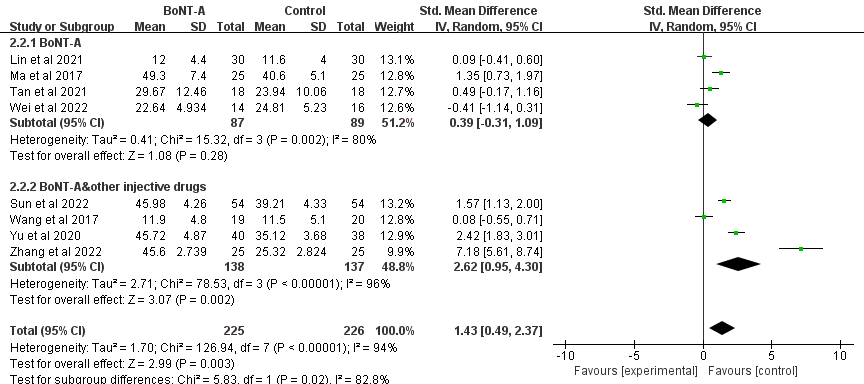

Supplement: Supplementary file 8 — Supplementary Material 8. Figure S6: Results of BoNT- A on UEFMA score in different intervention measures. [file 12891_2025_9347_MOESM8_ESM.png]

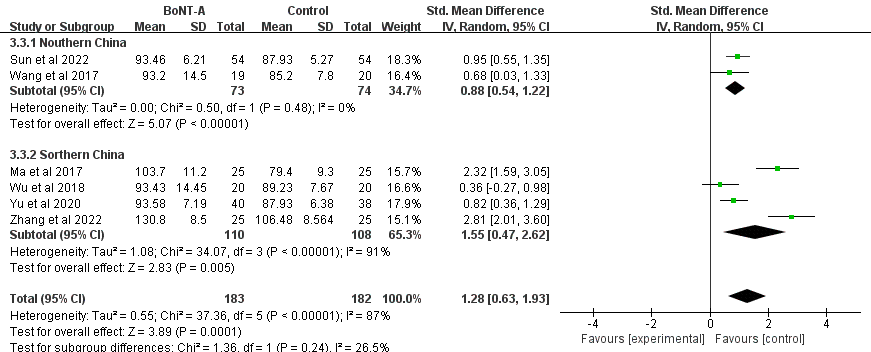

Supplement: Supplementary file 9 — Supplementary Material 9. Figure S7: Results of BoNT- A on flexion ROM in different geographic locations. [file 12891_2025_9347_MOESM9_ESM.png]

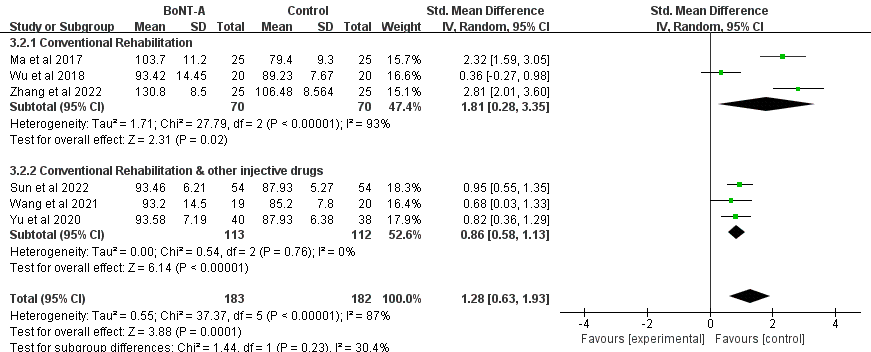

Supplement: Supplementary file 10 — Supplementary Material 10. Figure S8: Results of BoNT- A on flexion ROM in different control types. [file 12891_2025_9347_MOESM10_ESM.png]

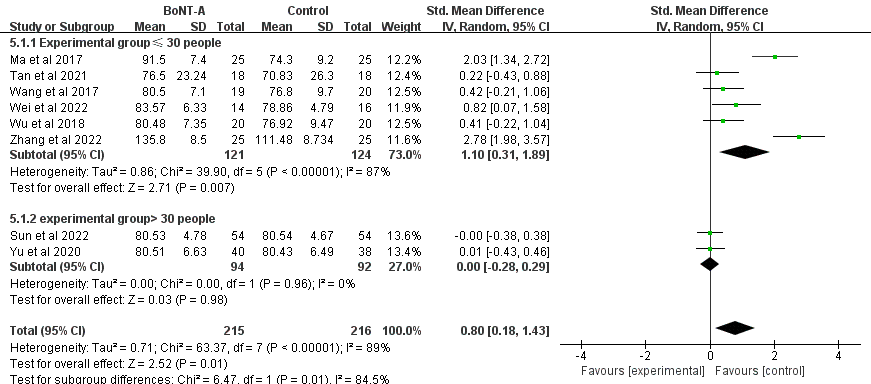

Supplement: Supplementary file 11 — Supplementary Material 11. Figure S9: Results of BoNT- A on abduction ROM with different sample sizes of people. [file 12891_2025_9347_MOESM11_ESM.png]

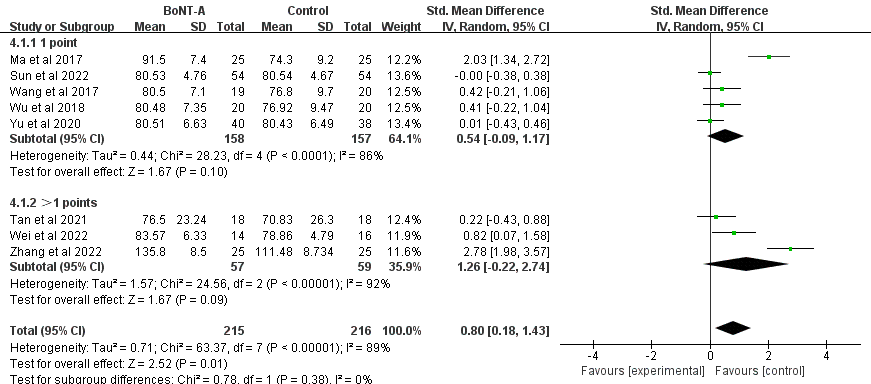

Supplement: Supplementary file 12 — Supplementary Material 12. Figure S10: Results of BoNT- A on abduction ROM in the different numbers of injection. [file 12891_2025_9347_MOESM12_ESM.png]

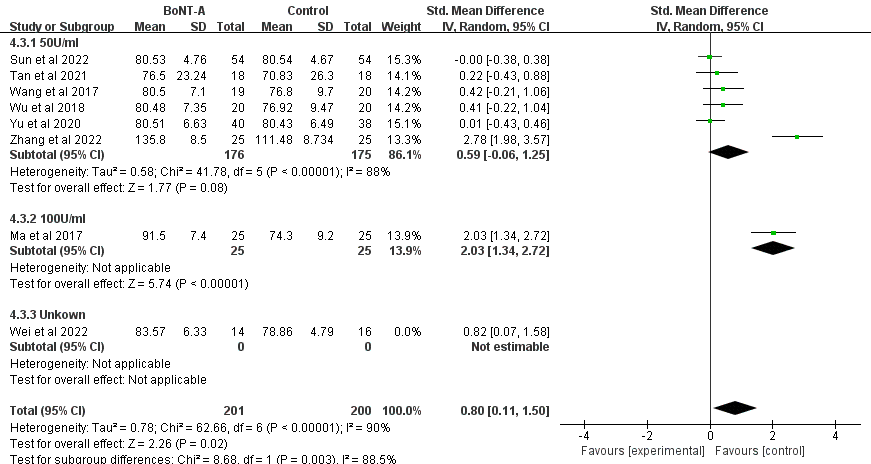

Supplement: Supplementary file 13 — Supplementary Material 13. Figure S11: Results of BoNT- A on abduction ROM in different dilution concentrations. [file 12891_2025_9347_MOESM13_ESM.png]

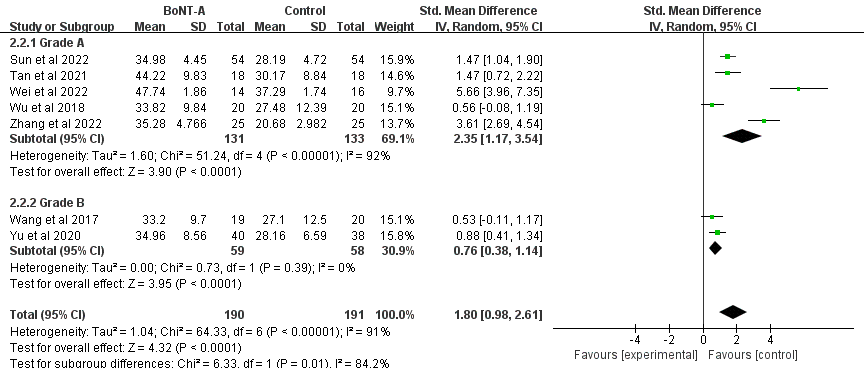

Supplement: Supplementary file 14 — Supplementary Material 14. Figure S12: Results of BoNT- A on external rotation ROM in different qualities of articles. [file 12891_2025_9347_MOESM14_ESM.png]

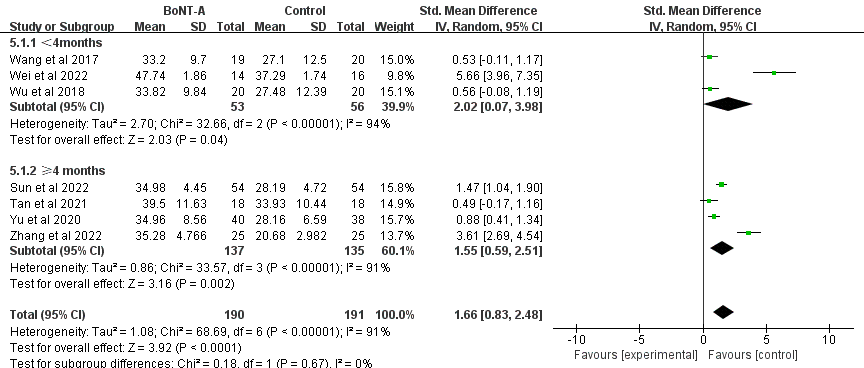

Supplement: Supplementary file 15 — Supplementary Material 15. Figure S13: Results of BoNT- A on external rotation ROM in different durations of disease. [file 12891_2025_9347_MOESM15_ESM.png]

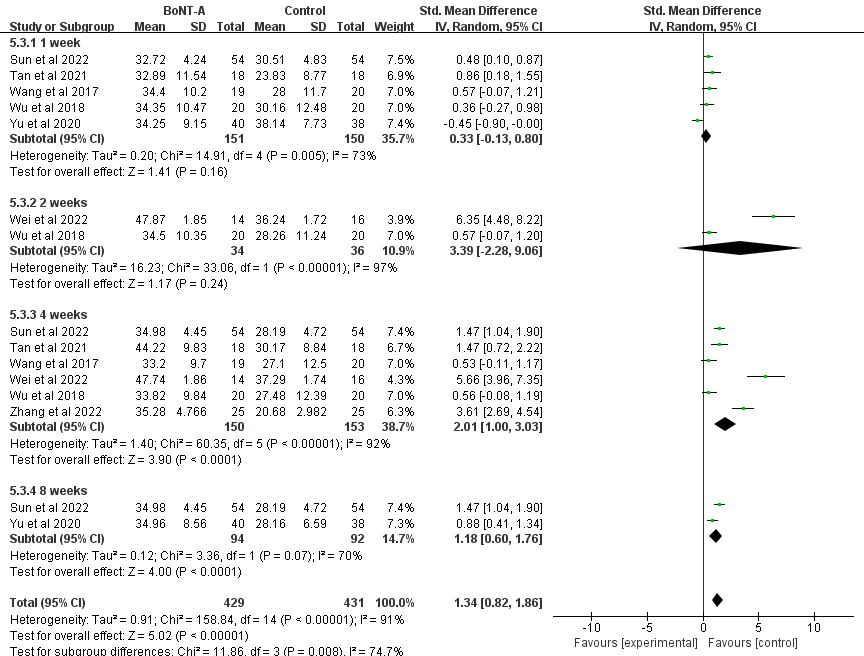

Supplement: Supplementary file 16 — Supplementary Material 16. Figure S14: Results of BoNT- A on external rotation in different follow-up periods. [file 12891_2025_9347_MOESM16_ESM.png]

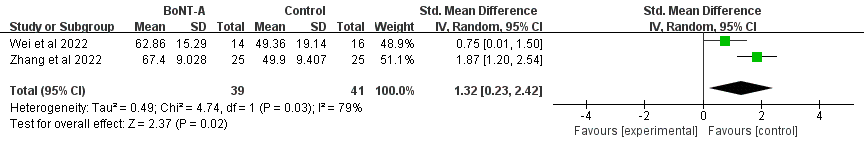

Supplement: Supplementary file 17 — Supplementary Material 17. Figure S15: Forest plot of the MBI score. [file 12891_2025_9347_MOESM17_ESM.png]

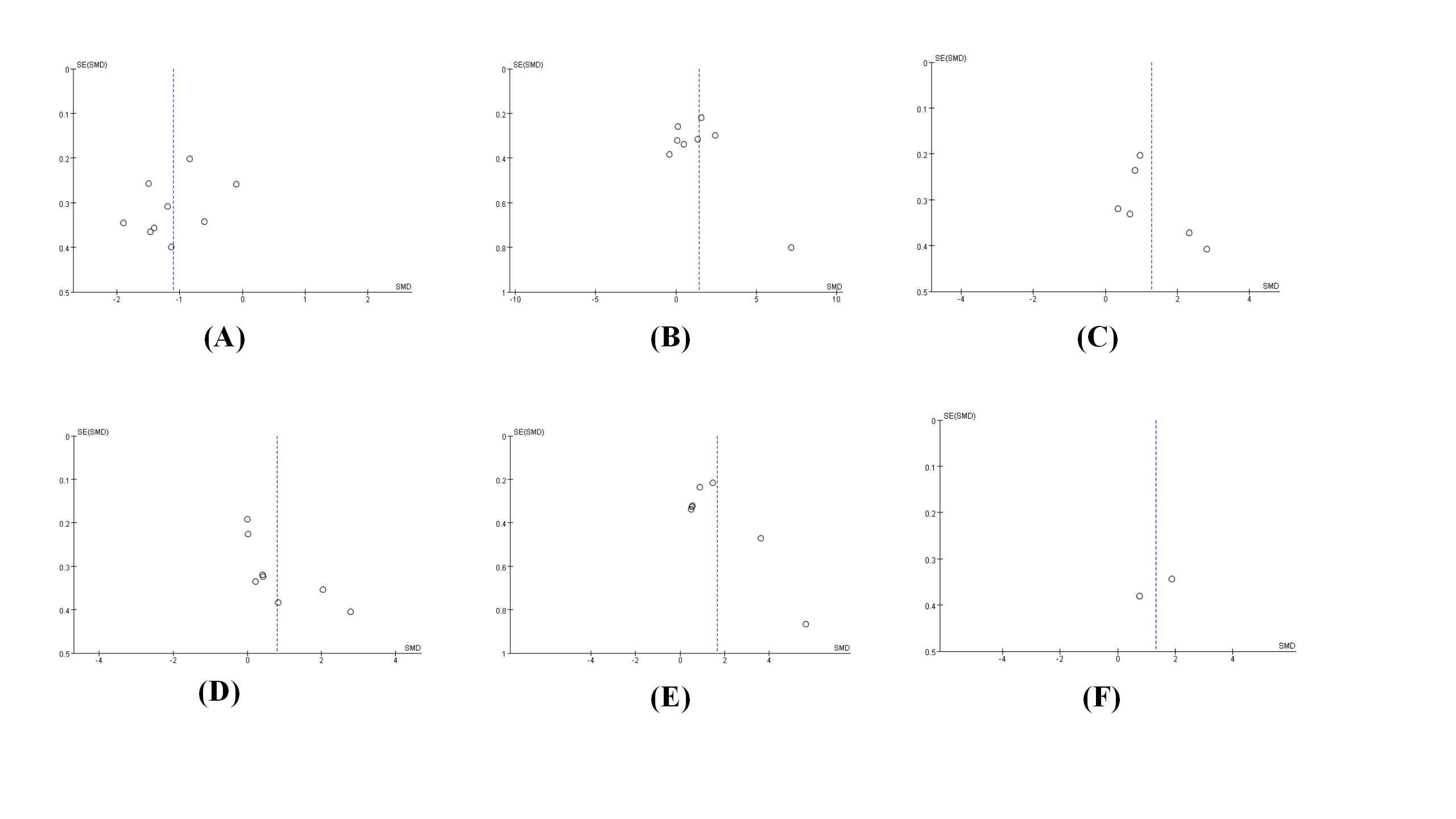

Supplement: Supplementary file 18 — Supplementary Material 18. Figure S16: Funnel plot of all outcomes. a (A) VAS score, (B) UEFMA score, (C) ROM of flexion, (D) ROM of abduction, (E) ROM of external rotation and (F) MBI score. [file 12891_2025_9347_MOESM18_ESM.png]

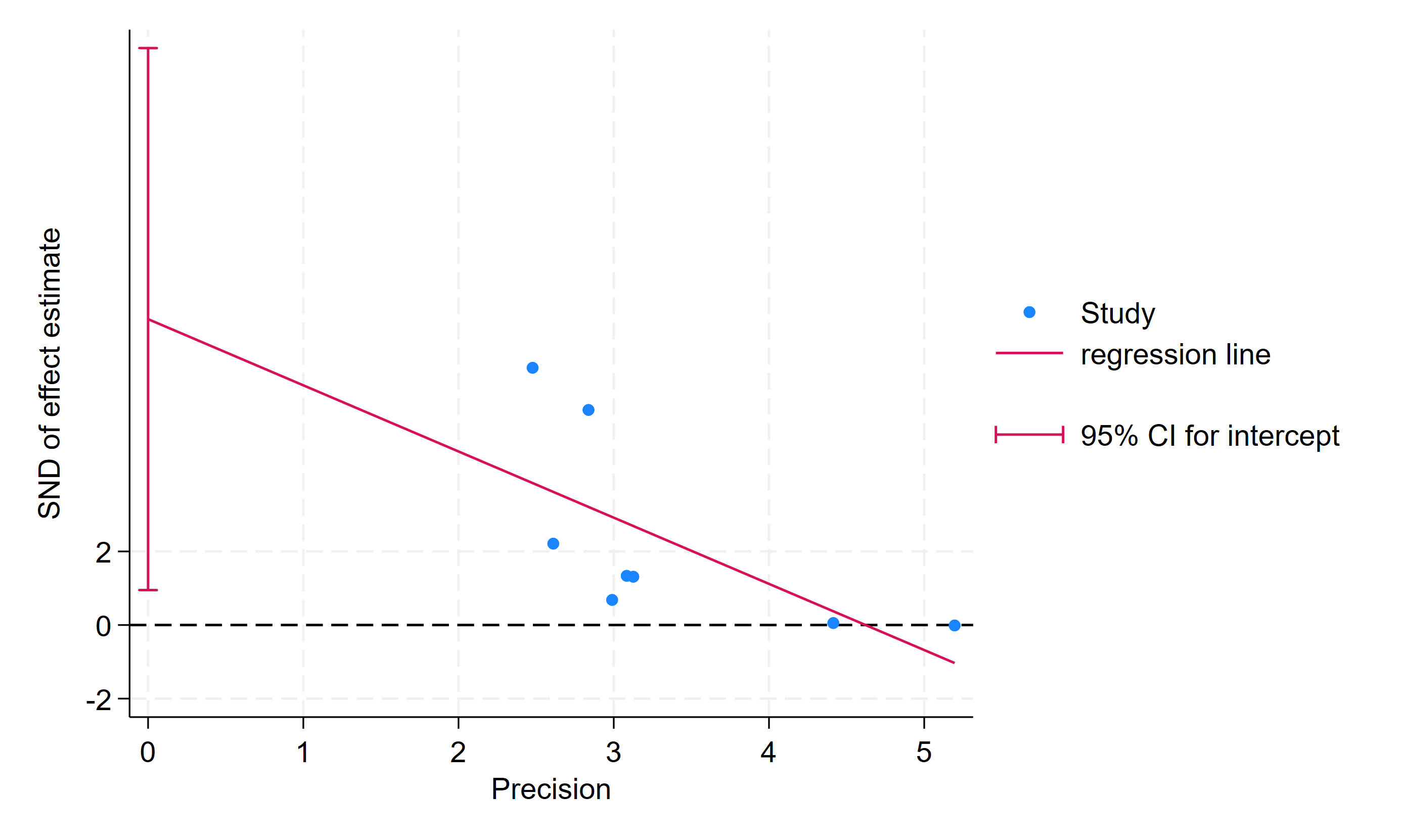

Supplement: Supplementary file 19 — Supplementary Material 19. Figure S17: Funnel plot with Egger's regression line for abduction ROM. [file 12891_2025_9347_MOESM19_ESM.png]

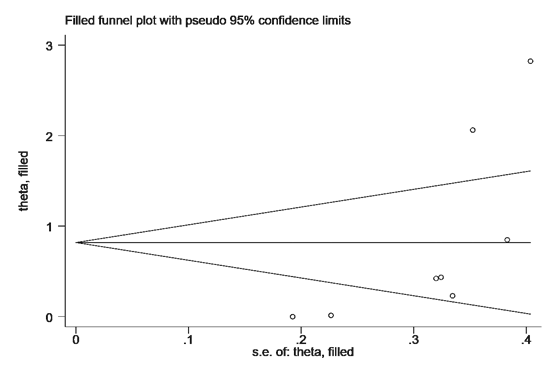

Supplement: Supplementary file 20 — Supplementary Material 20. Figure S18: Trim-and-fill adjusted plot for abduction ROM [file 12891_2025_9347_MOESM20_ESM.png]
